# Long-term health in individuals born preterm or with low birth weight: A cohort study

**DOI:** 10.1038/s41390-024-03346-6

**Published:** 2024-07-04

**Authors:** Coralie Amadou, Pierre-Yves Ancel, Jennifer Zeitlin, Céline Ribet, Marie Zins, Marie-Aline Charles

**Affiliations:** 1 Department of diabetes and endocrinology, Sud-Francilien Hospital, Corbeil-Essonnes (91 100), Paris-Saclay University, Orsay, France; 2 Université Paris Cité, INSERM, INRAE, Centre for Research in Epidemiology and Statistics (CRESS), Research team on early determinants of later health, EAROH, Paris, 75000 France; 3 Clinical Investigation Center CIC P1419, Assistance Publique-Hôpitaux de Paris, GH Paris Centre, Université Paris Cité, Paris, 75000 France; 4 Université Paris Cité, Paris Saclay University, UVSQ, Inserm UMS 011, Villejuif, France; 5 Université Paris Cité, Paris, 75000 France

## Abstract

**Background:**

To measure the association of prematurity and non-preterm low birth weight (LBW) with several long-term health outcomes.

**Methods:**

We selected adult participants from the Constances cohort. Associations between preterm birth (<37 weeks versus ≥37 weeks) and outcomes were measured using modified Poisson regression with adjustment for participant age and parental history. We used the same modeling methods to measure the association between LBW (i.e., <sex-specific 10th percentile) and outcomes in participants born ≥ 37 weeks. We tested for an interaction between exposures and sex.

**Results:**

Among 30,295 participants, preterm birth (5.2%) was associated with (RR[CI95]): obesity (1.25[1.08–1.46]), hypertriglyceridemia (1.23[1.07–1.42]), high LDL-cholesterol (1.16[1.05–1.28]), high blood pressure (HBP) (1.22[1.08–1.36]), metabolic syndrome (1.35[1.06–1.71]), non-alcoholic fatty liver disease (1.26[1.08–1.47]), allergic and atopic symptoms (1.06[1.01–1.12]), and lack of tertiary education (1.11[1.02–1.20]). Women had a significantly higher risk of hypertriglyceridemia and metabolic syndrome. In non-preterm participants, LBW was associated with prediabetes/diabetes (1.30[1.12–1.52]), HBP (1.22[(1.12–1.33]) and lack of tertiary education (1.13[1.07–1.20]), whereas the risk of obesity (0.83[0.73–0.95]) and abdominal obesity (0.84[0.76–0.93]) was reduced.

**Conclusion:**

Preterm birth and non-preterm LBW are both risk factors for several adult outcomes. However, regarding excess fat storage, their long-term effect seems to be in the opposite direction.

**Impact statement:**

- Preterm birth is associated with a higher long-term risk of obesity, whereas low birth weight is not.
- This study improves the understanding of the common idea that low birth weight is associated with a long-term risk of obesity, whereas it might depend on the cause of low birth weight.
- These findings provide new insights into the difficult distinction between the long-term adverse health effects of preterm birth and low birth weight.

## Introduction

Low birth weight (LBW), is a well-established risk factor for long-term health complications, especially cardiometabolic outcomes, in both children and adults.^1–5^ However, it is not always easy to distinguish the effects of prematurity from those of growth restriction because of the imprecision and sometimes missingness of gestational age data, particularly in studies involving subjects born in the early 20th century. Still, it is of great interest to study the long-term effects of prematurity in these adult subjects, who have the longest follow-up, as they remain controversial although several data support an excess risk of metabolic syndrome in individuals born preterm, including those with an appropriate for gestational age (AGA) birth weight.^6^

Regarding intra-uterine growth restriction, it is believed that the plasticity of early human development to adapt to an unfavorable environment, such as intrauterine undernutrition, can later lead to a mismatch where the adapted phenotype no longer matches the new environment and promote adverse conditions involving metabolic health.^7^

In a previous study, based on the French Constances cohort, we confirmed an excess risk of metabolic syndrome in individuals born with LBW (i.e., <10th percentile regardless of gestational age).^8^ In 2019, the Constances cohort participants received a new questionnaire requesting self-report information on their gestational age at birth. Based on these additional data, we aimed to assess the association between premature birth and long-term health in participants. As a secondary objective, we documented the long-term consequences of LBW in participants who were not born preterm. As for our previous study, we performed an outcome-wide analysis whose concept stands for the study of the association between a single exposure and multiple outcomes based on the same data set.^9^

## Methods

### Study population and data collection

The study included participants from the French population-based cohort “Constances”, a national sample of approximately 220,000 volunteers aged 18–69 years at baseline and living in 20 selected French “départements” (administrative divisions) in metropolitan France. The cohort design has been described previously, as has the data implementation and quality.^10,11^

This study accounted for participants included in the cohort from February 2012 to October 2020, regardless of whether they were born from singleton or multiple births. Data were mainly collected at the time of enrollment in the cohort through self-administered questionnaires, a physician-administered questionnaire, and clinical and paraclinical examinations performed in a medical center dedicated to the cohort. After baseline data collection, a supplemental self-administered questionnaire was sent to participants yearly to update baseline data or collect additional data. In addition, regular examinations were carried out in the health centers.

### Outcomes

The outcome-wide analysis included all chronic health conditions with a prevalence of at least 1% in the study population. We also studied the lack of tertiary education as a proxy of cognitive development. The definition and data source for each outcome are detailed in a supplemental table (Table S1). Most outcomes were collected at enrollment into the cohort, but some were collected through an additional questionnaire during follow-up.

### Birth term data collection and classification

Gestational age data were collected exclusively from a self-administered questionnaire sent to participants in 2019. Participants were asked to self-report their gestational age at birth from their health booklet (systematically provided at birth in France since 1945). In the questionnaire, a non-preterm birth was considered as a birth ≥ 37 weeks.

The health booklet has evolved over the years with advances in obstetrics, particularly in the estimation of gestational age with the development of ultrasound in the 1970s. Therefore, the presentation of data on gestational age at birth is dependent on the year of birth. Participants were then offered several response options to account for all versions of the health booklet, ranging from a binary response (preterm birth or not) to reporting an exact gestational age. Because of the variable precision of gestational age, with very few participants reporting their gestational age at birth on a week scale, we examined preterm birth as a single class (<37 weeks) and compared it with “non-preterm birth” (≥37 weeks) for its association with outcomes. The algorithm used to classify participants for preterm birth is provided in the Supplementary Appendix.

### Birth weight data collection and classification

Birth weight was recorded by a physician from the individual’s health booklet during a medical visit at enrollment into the cohort. In the analysis performed on individuals born ≥ 37 weeks, we defined LBW as birth weight below the 10th sex-specific percentile in the population of people born ≥ 37 weeks.

### Exclusion criteria

Individuals over the age of 60 were not included in the study due to the low availability of birth data. Participants with a birth weight <500 g or > 6000 g were excluded from the analyses as these values were considered likely to be unreliable.

To avoid misclassification regarding premature birth, we applied several exclusion criteria. If a participant responded inconsistently, gestational age data were considered missing. Outlier values (i.e., equivalent to <25 weeks or >42 weeks) were considered likely wrong and therefore classified as missing values. Gestational age values were considered inconsistent if the associated birth weight or length was <−5 or >+5 standard deviations of the values observed for the gestational age in the French population.^12^ For this data cleaning, we used the most detailed information on gestational age provided by the participant. Therefore, participants who reported a premature birth without further precision on gestational age were excluded from the analyses, as this clearance step could not be performed.

### Confounding factors

Following the principles of outcome-wide epidemiology,^13^ all models were adjusted for the same covariates in each multivariate analysis to compare relative risks between outcomes.

First, all models were adjusted for participants’ sex and age as they are associated with adult health and, theoretically, early life parameters. Indeed, we observed an association between age and the prevalence of preterm birth and extreme birth weights, probably resulting from the improvement of medical care related to these conditions in recent decades.

The other covariates were selected based on a priori knowledge and then confirmed by a univariate analysis of their associations with either premature birth or birth weight (as these exposures are strongly associated) with a p-value below 0.30.

To avoid selecting mediators rather than confounders, covariates had to precede pregnancy. Based on the data available in the Constances cohort, the covariates dataset included the geographical origin of the participant’s parents, the highest occupational category between the participant’s two parents, and the mother’s medical history.

The geographical origin of the participant’s parents was based on the country of birth and defined into four categories: Europe (including metropolitan France), Sub-Saharan Africa and French overseas departments and territories, Maghreb countries, and other geographical origins (including missing information).

The occupational category of the participant’s parents was defined into four categories: executive and intellectual professions (engineer, physician…); farmer, craftsman, shopkeeper, company director, and intermediate occupation (schoolteacher, nurse, social worker, technician, foreman, supervisor, etc.); employee (office or commercial employee, childminder, service agent, etc.), manual worker; homemaker, no occupation, or missing information.

Finally, the following covariates were examined in relation to the medical history of the participant’s mother: major adverse cardiovascular events (including history of stroke, myocardial infarction, or sudden death), high blood pressure, and diabetes. These covariates were taken into account regardless of the time of onset in relation to pregnancy because of imprecise or missing data on the age of onset in the participant’s mother. However, we made the reasonable assumption that these conditions could affect a pregnancy even several years before diagnosis due to early pathophysiological changes.

### Statistical analysis

Individuals’ characteristics are presented as mean with standard deviation for continuous variables and number and percentage for categorical variables. We described the characteristics of individuals excluded because of totally or partially missing data for birth data (supplemental material) and those finally included in the study.

Sex-specific susceptibility to the intrauterine environment has been described^14^ and the risk of most outcomes differs according to sex. Therefore, we performed separate analyses in men and women in addition to joint analyses. Differences between men and women were subsequently tested with an interaction test.

For the univariate analyses, the association between cofounders and premature birth was tested with a one-way ANOVA for continuous variables and a chi-square test (with continuity correction) for class variables. The association between birth term (preterm birth versus non-preterm birth, i.e., <37 weeks versus ≥37 weeks) and each outcome were analyzed with a modified Poisson regression using a robust error term to estimate relative risks^15^ with the GENMOD procedure from SAS.

Using the same procedure, we measured the association between LBW (i.e., <10th sex-specific percentile) and the outcomes in people born ≥37 weeks, aiming to document the effect of LBW apart from the context of prematurity.

All relative risks are reported with a 95% confidence interval. All analyses were performed using SAS version 9.4.

The data collected within the Constances cohort obtained authorization from the French National Commission for Information Technology and Liberties (CNIL) and the National Institute for Medical Research (Inserm) institutional review board. All studies using the Constances cohort data have received approval from a scientific committee. All participants have given written consent to use their data for scientific research.

Participants were not involved in the design, conduct, reporting, or dissemination plans of our research.

## Results

### Population selection and characteristics

**Fig. 1:**
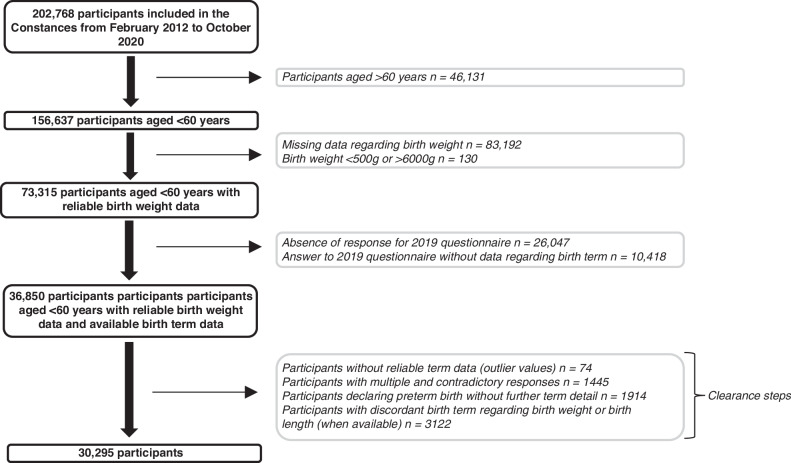
Flow chart.

The population selection is detailed in Fig. 1.

The Constances cohort included 202 768 participants from February 2012 to October 2020. After excluding participants older than 60 years and those without reliable birth weight data, 73,315 participants remained. Of these, 47,268 participants responded to the 2019 questionnaire, including 36,850 with a response regarding birth-term.

Only 12% of the participants who reported a non-preterm birth provided a detailed response on gestational age. In contrast, 94% of participants who reported a preterm birth provided a detailed response regarding gestational age. After excluding outliers, participants with inconsistent responses, participants with preterm birth but no further precision on gestational age, and participants with inconsistent gestational age with respect to birth weight or length, 30 295 participants had a reliable birth term status.

The characteristics of the participants selected for the study are described in Table 1. The characteristics of participants excluded from the study population because of missing data on birth weight and term are presented in the Supplementary Appendix (Table S2). In the study population (*n* = 30,295), the mean age (±SD) was 40 years (±9), 62% were women, and 33% had at least a master’s degree. The prevalence of preterm birth (i.e., <37 weeks) was 5.15% and was similar in men and women (5.07 and 5.20%, respectively).

**Table 1:** Characteristics of the study participants.

Population (*n*)	30,295
Age^a^: mean (SD)	39.62 (9.28)
Women *n* (%)	18,696 (61.7)
Birth weight, g (SD)	3323.41 (479.17)
Geographical origins of the mother *n* (%)	
Europe (including metropolitan France)	29,134 (96.2)
Maghreb countries	558 (1.8)
Sub-Saharan Africa and French overseas departments and territories	229 (0.8)
Other geographical origins or not available^b^	374 (1.2)
Geographical origins of the father *n* (%)	
Europe (including metropolitan France)	28,699 (94.7)
Maghreb countries	775 (2.6)
Sub-Saharan Africa and French overseas departments and territories	322 (1.1)
Other geographical origins or not available^b^	499 (1.6)
Highest parent’s occupation category *n* (%)	
Executive and intellectual professions	8379 (27.7)
Intermediate occupation	11,816 (39.0)
Employee or manual worker	9413 (31.1)
Other or not available^b^	687 (2.3)
Medical history of the mother *n* (%)	
Major cardiovascular event or sudden death	1390 (4.6)
Hypertension	5716 (18.9)
Diabetes	1712 (5.7)
Level of education of the participant (ISCED 2011^c^) *n* (%)	
Level 0 and 1 (early childhood and primary education)	232 (0.8)
Level 2 (lower secondary education)	453 (1.5)
Level 3 and 4 (upper and post-secondary education)	6660 (22.0)
Level 5 and 6 (short cycle tertiary education/Bachelor’s or equivalent)	12,654 (41.8)
Level 7 and 8 (Master’s/ Doctorate or equivalent)	10,006 (33.0)
Other	56 (0.2)
Not available	234 (0.8)

^a^Year of birth ranged from 1953 to 1999.

^b^Missing information regarding parent’s geographical origins represented approximately 40% of the “other or not available” category for the mother and 50% for the father.

Missing information regarding parental occupation represented less than 10% of the “other or not available” category for the mother and approximately 60% of the “other or not available” category for the father.

^c^*ISCED* International Standard Classification of Education.

### Association between preterm birth and long-term health

The distribution of exposures, covariates, and outcomes studied by preterm birth status is presented in Table 2. Preterm birth was associated with lower birth weight (2556 g versus 3 365 g), lower parental occupational category, and father’s geographical origin (higher prevalence of non-European countries). Regarding maternal health, only a history of major adverse cardiovascular events was significantly associated with preterm birth. Regarding the outcomes, compared with non-preterm birth, participants with preterm birth presented a significantly higher prevalence (from +2 to +4%) of obesity, abdominal obesity, hypertriglyceridemia, high LDL-cholesterol, high blood pressure, metabolic syndrome, non-alcoholic fatty liver disease, lack of tertiary education, and allergic and atopic symptoms. After adjustment for the participant’s age, the geographic origin of the participant’s parents, the highest occupational category between the participant’s two parents, and the mother’s medical history (including high blood pressure, diabetes, and major adverse cardiovascular event), the outcomes (except abdominal obesity) remained significantly associated with preterm birth. The results are shown in Table 3 for the whole population study and by sex. Cardiometabolic outcomes showed the highest excess risks in participants born < 37 weeks compared to those born ≥37 weeks with +35% for metabolic syndrome, +26% for non-alcoholic fatty liver disease, +25% for obesity, +23% for hypertriglyceridemia, +22% for high blood pressure, and +16% for high LDL cholesterol.

**Table 2:** Distribution of exposure, covariates, and studied outcomes by preterm birth status (univariate analysis).

*n*	Birth ≥ 37 weeks		Birth < 37 weeks		*p* value
	28 735		1 560		
**Age (mean (SD))**	39.58	(9.28)	40.31	(9.39)	0.002
**Women** ***n*** **(%)**	17724	(61.7)	972	(62.3)	0.639
**Birth weight (g) (mean (SD))**	3365.08	(437.77)	2555.83	(554.80)	<0.001
**Mother geographical origins** ***n*** **(%)**					0.217
Europe (including metropolitan France)	27649	(96.2)	1485	(95.2)	
Maghreb countries	522	(1.8)	36	(2.3)	
Sub-Saharan Africa and French overseas departments and territories	213	(0.7)	16	(1.0)	
Other or not available	351	(1.2)	23	(1.5)	
**Father geographical origins** ***n*** **(%)**					0.018
Europe (including metropolitan France)	27246	(94.8)	1453	(93.1)	
Maghreb countries	724	(2.5)	51	(3.3)	
Sub-Saharan Africa and French overseas departments and territories	296	(1.0)	26	(1.7)	
Other or not available	469	(1.6)	30	(1.9)	
**Parent’s highest occupational category** ***n*** **(%)**					0.041
Executive and intellectual professions	7951	(27.7)	428	(27.4)	
Intermediate occupation	11239	(39.1)	577	(37.0)	
Employee or manual worker	8907	(31.0)	506	(32.4)	
Other or not available	638	(2.2)	49	(3.1)	
**Maternal medical history** ***n*** **(%)**					
Major cardiovascular event or sudden death	1293	(4.5)	97	(6.2)	0.002
Hypertension	5408	(18.8)	308	(19.7)	0.382
Diabetes	1621	(5.6)	91	(5.8)	0.792
**Studied outcomes** ***n*** **(%)**					
Obesity	2339	(8.3)	163	(10.6)	0.002
Abdominal obesity	3843	(13.5)	240	(15.5)	0.027
Impaired fasting glucose/diabetes	1241	(4.4)	80	(5.3)	0.149
Hypertriglyceridemia	2492	(8.9)	171	(11.2)	0.003
High LDL-cholesterol	4541	(16.7)	301	(20.2)	<0.001
Low HDL-cholesterol	2306	(8.4)	135	(9.1)	0.410
High blood pressure	3590	(12.5)	246	(15.8)	<0.001
Metabolic syndrome	872	(3.2)	67	(4.5)	0.007
Non-alcoholic fatty liver disease	2064	(9.2)	147	(12.1)	0.001
Thyroid disease	1955	(6.9)	120	(7.8)	0.209
Asthma	2987	(10.6)	185	(12.1)	0.081
Sleep apnea syndrome	1117	(3.9)	54	(3.5)	0.434
History/symptoms of depression	6129	(21.3)	323	(20.7)	0.582
Anxiety symptoms	6014	(20.9)	333	(21.3)	0.717
Absence of tertiary education	6915	(24.3)	430	(27.8)	0.002
Unspecified inflammatory arthritis	254	(0.9)	16	(1.1)	0.663
Autoimmune inflammatory arthritis	384	(1.3)	21	(1.3)	1.000
Sciatica	4160	(14.5)	241	(15.4)	0.306
Allergic or atopic symptoms	12530	(43.6)	721	(46.2)	0.046
Psoriasis	1714	(6.0)	95	(6.1)	0.882
**Women only**					
Irregular periods	1716	(21.4)	90	(21.3)	0.998
Dysmenorrhea	3303	(41.9)	184	(44.3)	0.361
History of ART procedure	1374	(10.4)	73	(10.6)	0.930

**Table 3:** Multivariate analysis of the association between preterm birth (<37 versus ≥37 weeks) and outcomes.

	All (*n* = 30 295)			Men (*n* = 11 599)			Women (*n* = 18 696)		
	Relative Risk	CI95	*p* value	Relative Risk	CI95	*p* value	Relative Risk	CI95	*p* value
Obesity	1.25	(1.08–1.46)	0.003	1.24	(0.96–1.61)	0.1	1.25	(1.05–1.51)	0.01
Abdominal obesity	1.12	(0.99–1.26)	0.07	1.16	(0.90–1.48)	0.25	1.10	(0.97–1.26)	0.15
Impaired fasting glucose/diabetes	1.13	(0.91–1.40)	0.26	1.00	(0.73–1.38)	0.98	1.25	(0.93–1.68)	0.13
Hypertriglyceridemia^a^	1.23	(1.07–1.42)	0.005	1.03	(0.85–1.24)	0.78	1.58	(1.27–1.96)	<0.0001
High LDL-cholesterol	1.16	(1.05–1.28)	0.003	1.20	(1.05–1.38)	0.009	1.13	(0.98–1.30)	0.1
Low HDL-cholesterol	1.08	(0.91–1.27)	0.38	1.08	(0.88–1.33)	0.47	1.07	(0.82–1.39)	0.63
High blood pressure	1.22	(1.08–1.36)	<0.001	1.14	(0.97–1.32)	0.11	1.32	(1.11–1.57)	0.001
Metabolic syndrome^a^	1.35	(1.06–1.71)	0.01	1.01	(0.71–1.44)	0.96	1.86	(1.35–2.56)	<0.001
Non-alcoholic fatty liver disease	1.26	(1.08–1.47)	0.003	1.16	(0.96–1.41)	0.12	1.46	(1.14–1.87)	0.003
Thyroid disease	1.09	(0.91–1.29)	0.35	–			1.10	(0.92–1.31)	0.32
Asthma	1.14	(0.99–1.31)	0.07	1.08	(0.86–1.35)	0.51	1.18	(0.99–1.41)	0.07
Sleep apnea syndrome	0.85	(0.65–1.11)	0.24	0.70	(0.48–1.01)	0.06	1.09	(0.75–1.60)	0.65
History/symptoms of depression	0.95	(0.86–1.05)	0.36	0.99	(0.82–1.21)	0.95	0.94	(0.84–1.06)	0.3
Anxiety symptoms	1.01	(0.92–1.11)	0.82	1.05	(0.87–1.27)	0.6	1.00	(0.89–1.12)	0.96
Absence of tertiary education	1.11	(1.02–1.20)	0.01	1.10	(0.98–1.24)	0.12	1.11	(1.00–1.23)	0.05
Unspecified inflammatory arthritis	1.11	(0.67–1.82)	0.69	–			1.43	(0.84–2.45)	0.19
Autoimmune inflammatory arthritis	0.96	(0.62–1.48)	0.86	–			1.16	(0.73–1.86)	0.53
Sciatica	1.05	(0.93–1.19)	0.4	0.90	(0.71–1.15)	0.41	1.12	(0.97–1.28)	0.12
Allergic or atopic symptoms^a^	1.06	(1.01–1.12)	0.03	1.15	(1.05–1.26)	0.002	1.02	(0.95–1.09)	0.65
Psoriasis	1.02	(0.84–1.25)	0.82	0.95	(0.68–1.32)	0.75	1.07	(0.84–1.38)	0.58
Women’s health									
Irregular periods							0.99	(0.82–1.20)	0.94
Dysmenorrhea							1.06	(0.95–1.18)	0.33
History of ART procedure							1.01	(0.81–1.26)	0.93

Adjustment for the participant’s sex, age, the geographical origin of the participant’s parents, the highest occupational category between the two parents of the participant and the medical history of the mother (including HBP, diabetes, and MACE).

As the number of cases of thyroid disease, unspecified inflammatory arthritis and autoimmune inflammatory arthritis was insufficient in men to perform analyses, these outcomes were studied only in women in analyses by sex.

^a^Significant interaction between sex and preterm birth.

Regarding sex differences, interaction tests confirmed a significant difference between men and women only for hypertriglyceridemia and metabolic syndrome (higher excess risk and significant only in women) and for allergic and atopic symptoms (higher excess risk and significant only in men).

### Association between non-preterm low birth weight and long-term health

The distribution of exposures, covariates, and outcomes studied according to birth weight category in non-preterm participants are presented in Table 4 (univariate analyses). LBW (i.e., < 10th versus > 10th sex-specific percentile) was associated with sex (higher percentage of women), maternal geographical origins (higher prevalence of non-European countries) and lower parental occupational category. Regarding maternal health, a history of major adverse cardiovascular events and high blood pressure were significantly associated with LBW.

**Table 4:** Distribution of exposures, covariates, and studied outcomes by birth weight in people born after 37 weeks (univariate analysis).

n	Birth weight > 10th sex-specific percentile		Birth weight < 10th sex-specific percentile		*p* value
	25,685		3050		
**Age (mean (SD))**	39.59	(9.26)	39.55	(9.43)	0.843
**Women** ***n*** **(%)**	15792	(61.5)	1932	(63.3)	0.048
**Birth weight (g) (mean (SD))**	3451.10	(372.14)	2640.65	(228.52)	<0.001
**Mother geographical origins** ***n*** **(%)**					0.022
Europe (including metropolitan France)	24,732	(96.3)	2917	(95.6)	
Maghreb countries	471	(1.8)	51	(1.7)	
Sub-Saharan Africa and French overseas departments and territories	182	(0.7)	31	(1.0)	
Other or not available	300	(1.2)	51	(1.7)	
**Father geographical origins** ***n*** **(%)**					0.061
Europe (including metropolitan France)	24,369	(94.9)	2877	(94.3)	
Maghreb countries	654	(2.5)	70	(2.3)	
Sub-Saharan Africa and French overseas departments and territories	257	(1.0)	39	(1.3)	
Other or not available	405	(1.6)	64	(2.1)	
**Parent’s highest occupational category** ***n*** **(%)**					0.009
Executive and intellectual professions	7145	(27.8)	806	(26.4)	
Intermediate occupation	10,092	(39.3)	1147	(37.6)	
Employee or manual worker	7882	(30.7)	1025	(33.6)	
Other or not available	566	(2.2)	72	(2.4)	
**Maternal medical history** ***n*** **(%)**					
Major cardiovascular event or sudden death	1123	(4.4)	170	(5.6)	0.003
Hypertension	4781	(18.6)	627	(20.6)	0.010
Diabetes	1470	(5.7)	151	(5.0)	0.088
**Studied outcomes** ***n*** **(%)**					
Obesity	2125	(8.4)	214	(7.1)	0.018
Abdominal obesity	3489	(13.7)	354	(11.7)	0.003
Impaired fasting glucose/diabetes	1076	(4.3)	165	(5.6)	0.002
Hypertriglyceridemia	2210	(8.8)	282	(9.5)	0.237
High LDL-cholesterol	4040	(16.6)	501	(17.3)	0.342
Low HDL-cholesterol	2060	(8.4)	246	(8.5)	0.975
High blood pressure	3131	(12.2)	459	(15.1)	<0.001
Metabolic syndrome	768	(3.2)	104	(3.6)	0.208
Non-alcoholic fatty liver disease	1864	(9.3)	200	(8.5)	0.199
Thyroid disease	1752	(7.0)	203	(6.8)	0.748
Asthma	2657	(10.6)	330	(11.1)	0.425
Sleep apnea syndrome	1001	(3.9)	116	(3.8)	0.838
History/symptoms of depression	5498	(21.4)	631	(20.7)	0.382
Anxiety symptoms	5332	(20.8)	682	(22.4)	0.042
Absence of tertiary education	6081	(23.9)	834	(27.7)	<0.001
Unspecified inflammatory arthritis	228	(0.9)	26	(0.9)	0.919
Autoimmune inflammatory arthritis	335	(1.3)	49	(1.6)	0.197
Sciatica	3698	(14.4)	462	(15.1)	0.278
Allergic or atopic symptoms	11191	(43.6)	1339	(43.9)	0.742
Psoriasis	1530	(6.0)	184	(6.0)	0.899
**Women only**					
Irregular periods	1523	(21.3)	193	(22.7)	0.361
Dysmenorrhea	2956	(42.0)	347	(41.6)	0.843
History of ART procedure	1229	(10.5)	145	(10.1)	0.690

Regarding the outcomes, LBW was significantly associated with a higher prevalence (from +1 to +3%) of impaired fasting glucose or diabetes, high blood pressure, anxiety symptoms, and absence of tertiary education. On the other hand, LBW was associated with a lower prevalence of obesity (−1.3%) and abdominal obesity (−2%).

In multivariate analyses, the outcomes that showed a significant excess risk in those born with LBW were impaired fasting glucose/diabetes (+30%), high blood pressure (+22%), and lack of tertiary education (+13%), in contrast to obesity and abdominal obesity, for which those born with LBW showed a risk reduction (−17% and −16%, respectively). Regarding sex differences, interaction tests confirmed a significant difference only for impaired fasting glucose/diabetes (higher excess risk and significant only in men). These analyses are shown in Table 5.

**Table 5:** Multivariate analysis of the association between low birth weight ( < 10^th^ sex-specific percentile versus ≥ 10^th^ sex-specific percentile) in people born after 37 weeks.

	All (*n* = 28,735)			Men (*n* = 11,011)			Women (*n* = 17,724)		
	Relative Risk	CI95	*p* value	Relative Risk	CI95	*p* value	Relative Risk	CI95	*p* value
Obesity	0.83	(0.73–0.95)	0.007	0.81	(0.63–1.03)	0.08	0.84	(0.72–0.99)	0.04
Abdominal obesity	0.84	(0.76–0.93)	<0.001	0.81	(0.65–1.00)	0.05	0.84	(0.75–0.95)	0.003
Impaired fasting glucose/diabetes^a^	1.30	(1.12–1.52)	<0.001	1.53	(1.25–1.88)	<0.0001	1.08	(0.85–1.37)	0.53
Hypertriglyceridemia	1.09	(0.97–1.22)	0.14	1.13	(0.98–1.30)	0.09	1.02	(0.83–1.25)	0.85
High LDL-cholesterol	1.04	(0.96–1.13)	0.32	1.10	(0.99–1.23)	0.09	0.99	(0.88–1.11)	0.86
Low HDL-cholesterol	1.02	(0.90–1.15)	0.79	1.02	(0.87–1.20)	0.79	1.01	(0.82–1.23)	0.95
High blood pressure	1.22	(1.12–1.33)	<0.0001	1.22	(1.09–1.36)	<0.001	1.23	(1.07–1.40)	0.003
Metabolic syndrome	1.14	(0.93–1.39)	0.2	1.22	(0.95–1.57)	0.11	1.03	(0.74–1.42)	0.88
Non-alcoholic fatty liver disease	0.91	(0.80–1.05)	0.19	0.99	(0.84–1.16)	0.86	0.80	(0.63–1.02)	0.08
Thyroid disease	0.96	(0.83–1.10)	0.53	–			0.95	(0.83–1.10)	0.52
Asthma	1.04	(0.93–1.16)	0.46	1.00	(0.85–1.19)	0.96	1.07	(0.93–1.23)	0.36
Sleep apnea syndrome	0.98	(0.82–1.19)	0.86	0.91	(0.71–1.16)	0.43	1.10	(0.83–1.47)	0.51
History/symptoms of depression	0.95	(0.89–1.03)	0.2	1.01	(0.87–1.17)	0.88	0.94	(0.86–1.02)	0.12
Anxiety symptoms	1.07	(0.99–1.14)	0.08	1.04	(0.90–1.20)	0.57	1.08	(0.99–1.16)	0.08
Absence of tertiary education	1.13	(1.07–1.20)	<0.0001	1.12	(1.02–1.23)	0.01	1.14	(1.05–1.23)	0.001
Unspecified inflammatory arthritis	0.94	(0.63–1.41)	0.77	–			0.89	(0.54–1.46)	0.65
Autoimmune inflammatory arthritis	1.20	(0.89–1.62)	0.22	–			1.19	(0.84–1.69)	0.33
Sciatica	1.04	(0.95–1.14)	0.4	0.97	(0.81–1.15)	0.71	1.07	(0.96–1.18)	0.21
Allergic or atopic symptoms	1.00	(0.96–1.05)	0.86	1.00	(0.93–1.08)	0.99	1.00	(0.95–1.06)	0.86
Psoriasis	1.01	(0.87–1.17)	0.88	0.96	(0.75–1.23)	0.76	1.04	(0.86–1.25)	0.69
Women’s health									
Irregular periods	1.06	(0.93–1.21)	0.39				1.06	(0.93–1.21)	0.39
Dysmenorrhea	0.98	(0.90–1.06)	0.61				0.98	(0.90–1.06)	0.61
History of ART procedure	0.96	(0.82–1.14)	0.66				0.96	(0.82–1.14)	0.66

Adjustment for the participant’s sex, age, the geographical origin of the participant’s parents, the highest occupational category between the two parents of the participant, and the medical history of the mother (including HBP, diabetes, and MACE).

As the number of cases of thyroid disease, unspecified inflammatory arthritis and autoimmune inflammatory arthritis was insufficient in men to perform analyses, these outcomes were studied only in women in analyses by sex.

^a^Significant interaction between sex and birth weight.

## Discussion

The aim of this study was to document the health status and educational attainment of young adults born preterm in a contemporary French cohort. In addition, we attempted to disentangle the outcomes associated with immaturity at birth from those associated with low birth weight outside the context of prematurity. We found several outcomes, mainly obesity and metabolic impairment, associated with preterm birth. Finally, the analysis restricted to non-preterm participants showed that people born with LBW also have an excess risk for metabolic impairment but a reduced risk for obesity and abdominal obesity. Therefore, these results support the same direction of association for exposures to preterm birth and LBW, with an important exception for excess fat storage, which may be specifically associated with long-term outcomes of prematurity.

However, before drawing any conclusions, it is important to mention the limitations of this study. The most important limitation is the lack of precision of gestational age. Very few participants could give a gestational age in weeks, and those who did were the youngest, with a lower prevalence of the health conditions we studied. Therefore, we could only consider preterm status as a binary variable. In addition, our population included only people who survived the consequences of prematurity and reached adulthood. This survival bias adds to the selection biases of consent to participate in a cohort and availability of the birth health booklet, which is associated with a higher level of education, as shown in the Supplementary Appendix (Table S2). We then assume that individuals with extreme prematurity and subsequent poorer mental health and educational outcomes were less likely to participate in the study. This may have led to an underestimation of the long-term effects of preterm birth.

However, our study has several strengths. The outcome-wide design allowed us to describe the overall health burden of preterm birth in the second half of the 20th century for people who reached adulthood. The analysis is based on reliable data on birth weight, preterm birth, medical assessment for several outcomes, and biological parameters. We were able to adjust for familial socioeconomic confounders and maternal medical history. This latter adjustment was to account for shared genetic factors between prematurity and outcomes that could confound the observed associations (although this adjustment had almost no effect on the strength of the associations with metabolic outcomes). Finally, the main originality stems from the outcome-wide design, which allows comparison of the effect size between one exposure and multiple outcomes at the same time and in the same population.

Overall, our findings are consistent with previous literature related to long-term outcomes of being born preterm or LBW.^16^ Therefore, we offer to focus the discussion on the most original finding of our study, i.e., the opposite association between both obesity and non-alcoholic fatty liver disease in relation to exposure to preterm birth (excess risk) and non-preterm LBW (reduced risk). In contrast, both exposures were associated with an excess risk of metabolic outcomes. First, this finding goes against the common idea of an association between LBW and obesity. Indeed, the association between LBW and long-term risk of obesity has never been clearly established, and some evidence tends to suggest a reduced risk.^17,18^ This confusion may come from the cause of LBW, as people with LBW resulting from prematurity might present a higher risk of obesity in later life as opposed to people with LBW resulting from intra-uterine growth restriction (small for gestational age). However since prematurity and LBW can sometimes share the same causes, pathways leading to obesity are very complex to understand.

Regarding prematurity and long-term risk of obesity, a recent meta-analysis concluded to a significant association between childhood and adolescence.^19^ In the same analysis, as previously reported, accelerated postnatal weight gain was associated with obesity. This meta-analysis showed no difference in the risk of obesity between those born preterm with SGA or AGA. However, the odds ratios indicated, though nonsignificant, a possible risk reduction for SGA compared with AGA, in line with our results. However, this sub-analysis was limited to only four studies with fewer participants than ours. Another individual participant data meta-analysis of 16 birth cohorts involving more than 250,000 children reported lower gestational age and preterm birth were associated with lower BMI Z-score in early childhood.^20^ The association disappears in late adolescence, while a trend for a higher risk of being overweight emerges during this period. An increased fat mass storage with age, especially through accelerated weight gain in later childhood, may later explain an excess risk of obesity in adolescents and adults born preterm.^21^ Regarding individuals with non-preterm LBW, the excess risk of high blood pressure and diabetes, despite a significantly reduced risk of obesity that we observed in our study, advocates for complex insulin resistance mechanisms in these people. Apart from early weight catch-up and specific body composition, pathophysiology could involve the liver and muscles’ insulin sensitivity. On the other hand, a diminution of the number of beta cells and nephrons, which contribute to the risk of diabetes and high blood pressure later in life, has been observed in animals in the context of intra-uterine growth restriction.^22,23^ However, there is a lack of large-scale prospective studies investigating non-obese individuals with a history of non-premature LBW. Still, our findings correlate with those of a French prospective cohort that confirmed a higher prevalence of metabolic syndrome in adults born non-preterm with SGA, even after adjustment for BMI.^24^

Finally, regarding sex differences, interaction tests were not significant for most outcomes. However, they revealed a higher excess risk for some metabolic outcomes in preterm women compared with preterm men. Such differences between sexes have already been identified in several studies.^25^ One may hypothesize a selective survival bias as previous findings supported an excess morbidity and mortality in male infant,^26^ as confirmed by nationwide data like those of the Swedish national birth registry.^27^ Therefore, the ‘cost’ of female fetus advantage in neonatal outcomes, through a better hormonal profile and adaptation to oxidative stress, could be the increase of long-term adverse effects.^28^ However, our results concerning the higher risk for diabetes in men with non-preterm LBW compared to women in the same situation challenges this hypothesis. The different susceptibility of men and women to these two different early life situations needs confirmation in other studies.

## Conclusion

This study confirms previous findings of an excess risk of several poor health outcomes in people born preterm. It provides a global view of the health status of individuals born preterm in the second half of the 20th century. However, it also mitigates the common idea of an association between LBW and obesity. Obesity in adulthood appears specifically related to preterm birth since we found the inverse association in people born non-preterm with LBW.

## Supplementary information

Supplementary material

Table S1

## Data Availability

French health data regulations protect data from this study (French National Commission on Informatics and Liberty “Commission Nationale de l’Informatique et des Libertés”). Data can be made available upon reasonable request to the steering committee of the CONSTANCES cohort study after legal verification of the use of the data.
