# Supplementary material for: Long-term health in individuals born preterm or with low birth weight: A cohort study
Source: Pediatr Res. 2024 Jul 4;97(2):577–85. doi: 10.1038/s41390-024-03346-6 (PMC12015107; doi:10.1038/s41390-024-03346-6)
Supplement: Supplementary file 1 — Supplementary material [file 41390_2024_3346_MOESM1_ESM.pdf]

Algorithm used to classify the participants regarding premature birth according to the information obtained from the Health Booklet:

Participants were asked to respond to at least one of the four following items (pregnancy duration considered starting from the first day of the last menstrual period):

1) *“Were you born full term (i.e., ≥ 8.5 months)?”* Accepted responses (checkboxes) were: *“yes”*, *“no”*, or *“I don’t know”*.
2) “*What was your birth term in months?*” Admitting one decimal.
3) *“How many days before the due date were you born?”* Considering the due date is calculated at +41 weeks from the first day of last menstrual period.
4) *“What was your birth term in weeks of pregnancy?”*

Based on the answers to this questionnaire, and in line with the WHO definitions, we classify participants in 4 categories: “full-term birth”, “preterm birth without precision”, “moderate to late preterm birth”, and “very to extremely preterm birth”.

If participants answered item 1:

- *“I don’t know”*: data was considered missing.
- *“Yes”*: the participant was considered “full-term birth”.
- *“No”*: the participant was considered “preterm birth without precision”.

If participants answered item 2, he/she was classified among: “full-term birth” if the birth term was ≥ 8.5 months, preterm otherwise “moderate to late preterm birth” if the birth term was from 7.4 to 8.4 months, “very to extremely preterm birth” if the birth term was <7.4 months.

If participants answered item 3 (number of days before the due date, considering a due date at +41 weeks of pregnancy), he/she was classified among: “full-term birth” if the number of days was ≤ 28 days before the due date, preterm otherwise “moderate to late preterm birth” if the number of days was from 29 to 63 days before the due date, “very to extremely preterm birth” if the number of days was > 63 days before the due date.

If participants answered item 4 (weeks of pregnancy), he/she was classified among: “full-term birth” if the birth term was ≥ 37 weeks, preterm otherwise “moderate to late preterm birth” if

the birth term was from 32 weeks to 36 weeks+ 6 days, “very to extremely preterm birth” if the birth term was <32 weeks.

If participants answered multiple items, the most specific answer was retained in the following priority order (from the most to less specific): item 4 (weeks of pregnancy) > item 3 (days before due date) > item 2 (months of pregnancy) > item 1 (yes/no question).

Table S2. Characteristics of the Constance cohort participants (< 60 years) excluded from the study because of missing data regarding birth weight ± term.

	Participants without reliable birth weight data	Participants with reliable birth weight data but without birth term data
Population (n)	83 322	43 020
Age: mean (SD)	45.4 (10.3)	36.53 (9.38)
Women (%)	43063 (51.7)	23415 (54.4)
Birth weight, g (SD)		3296.64 (496.40)
Geographical origins of the mother (%)		
Europe (including metropolitan France)	70504 (84.6)	40005 (93.0)
Maghreb countries	5079 (6.1)	1392 (3.2)
Sub-Saharan Africa and French overseas departments and territories	2919 (3.5)	728 (1.7)
Other geographical origins or not available	4820 (5.8)	895 (2.1)
Geographical origins of the father (%)		
Europe (including metropolitan France)	69365 (83.2)	39317 (91.4)
Maghreb countries	5648 (6.8)	1765 (4.1)
Sub-Saharan Africa and French overseas departments and territories	3054 (3.7)	804 (1.9)
Other geographical origins or not available	5255 (6.3)	1134 (2.6)
Highest parent's occupation category (%)		
Executive and intellectual professions	18114 (21.7)	11182 (26.0)
Intermediate occupation	31408 (37.7)	15655 (36.4)
Employee or manual worker	29887 (35.9)	14844 (34.5)
Other or not available	3913 (4.7)	1339 (3.1)
Medical history of the mother (%)		
Major cardiovascular event or sudden death	6357 (7.6)	1766 (4.1)
Hypertension	17397 (20.9)	7341 (17.1)
Diabetes	7610 (9.1)	2515 (5.8)
Level of education (ISCED 2011) (%)		
Level 0 and 1 (early childhood and primary education)	3320 (4.0)	661 (1.5)
Level 2 (lower secondary education)	3943 (4.7)	882 (2.1)
Level 3 and 4 (upper and post-secondary education)	27963 (33.6)	11938 (27.7)
Level 5 and 6 (short cycle tertiary education/Bachelor's or equivalent)	27225 (32.7)	16625 (38.6)
Level 7 and 8 (Master's/ Doctorate or equivalent)	19117 (22.9)	12402 (28.8)
Other	230 (0.3)	82 (0.2)
Not available	1524 (1.8)	430 (1.0)
